# A RETROSPECTIVE COHORT STUDY OF HOSPITAL DISCHARGE INSTRUCTIONS FOLLOWING DELIRIUM EPISODES

**DOI:** 10.1093/geroni/igae098.2227

**Published:** 2024-12-31

**Authors:** Blair Golden, David Sonnentag, Farah Kaiksow, Andrea Gilmore-Bykovskyi, Manish Shah, Sharon Inouye, Eduard Vasilevskis

**Affiliations:** University of Wisconsin Madison School of Medicine and Public Health, Madison, Wisconsin, United States; University of Wisconsin School of Medicine and Public Health, Madison, Wisconsin, United States; University of Wisconsin School of Medicine and Public Health, Madison, Wisconsin, United States; University of Wisconsin Madison, Madison, Wisconsin, United States; University of Wisconsin School of Medicine & Public Health, Madison, Wisconsin, United States; Harvard Medical School, Boston, Massachusetts, United States; University of Wisconsin Madison, Madison, Wisconsin, United States

## Abstract

Delirium impacts an estimated 25% of hospitalized older adults and is associated with adverse outcomes, including increased mortality. Delirium can persist or recur after discharge, and prompt recognition is critical. Caregiver education may improve delirium recognition and management in the post-acute care period, but it is unclear if delirium is routinely incorporated into hospital discharge instructions. We sought to characterize how clinicians communicate about delirium in written discharge instructions. We performed a retrospective chart review of a random sample of 50 hospitalized older adults (age ≥65) with delirium. Trained team members identified delirium diagnoses from discharge summaries and assessed all mentions of delirium within discharge instructions. We also identified new anti-psychotic or benzodiazepine prescriptions and neurology or geriatrics referrals, as these are routinely communicated at discharge and may represent indirect delirium instruction. Almost all (48, 96%) patients had personalized discharge instructions regarding at least one non-delirium hospital diagnosis, but only 2 cases (4%) had delirium included in discharge instructions. A quarter of instructions (13, 26%) had terms that could be considered synonyms for delirium (e.g., “confusion” (5), “altered mental status” (5)). Three cases (6%) had proxies for delirium instruction (new prescriptions or clinic referrals). Documentation trends were similar for populations from whom delirium instructions would be particularly important, including patients discharging home and persons living with dementia. These findings demonstrate a large gap between clinician recognition of delirium and instructions to patients and caregivers during a vulnerable transition period. Future work is critical to understand discharge educational needs surrounding delirium.

